# *In Silico* and Structural Analyses Demonstrate That Intrinsic Protein Motions Guide T Cell Receptor Complementarity Determining Region Loop Flexibility

**DOI:** 10.3389/fimmu.2018.00674

**Published:** 2018-04-11

**Authors:** Christopher J. Holland, Bruce J. MacLachlan, Valentina Bianchi, Sophie J. Hesketh, Richard Morgan, Owen Vickery, Anna M. Bulek, Anna Fuller, Andrew Godkin, Andrew K. Sewell, Pierre J. Rizkallah, Stephen Wells, David K. Cole

**Affiliations:** ^1^Division of Infection and Immunity and Systems Immunity Research Institute, Cardiff University School of Medicine, Cardiff, United Kingdom; ^2^Immunocore, Abingdon, United Kingdom; ^3^Department of Oncology, University Hospital of Lausanne, Lausanne, Switzerland; ^4^Astbury Centre for Structural Molecular Biology, University of Leeds, Leeds, United Kingdom; ^5^Department of Chemistry, University of Bath, Bath, United Kingdom

**Keywords:** T-cells, T cell receptor, complementarity determining regions loops, protein flexibility, computational simulations, X-ray crystallography

## Abstract

T-cell immunity is controlled by T cell receptor (TCR) binding to peptide major histocompatibility complexes (pMHCs). The nature of the interaction between these two proteins has been the subject of many investigations because of its central role in immunity against pathogens, cancer, in autoimmunity, and during organ transplant rejection. Crystal structures comparing unbound and pMHC-bound TCRs have revealed flexibility at the interaction interface, particularly from the perspective of the TCR. However, crystal structures represent only a snapshot of protein conformation that could be influenced through biologically irrelevant crystal lattice contacts and other factors. Here, we solved the structures of three unbound TCRs from multiple crystals. Superposition of identical TCR structures from different crystals revealed some conformation differences of up to 5 Å in individual complementarity determining region (CDR) loops that are similar to those that have previously been attributed to antigen engagement. We then used a combination of rigidity analysis and simulations of protein motion to reveal the theoretical potential of TCR CDR loop flexibility in unbound state. These simulations of protein motion support the notion that crystal structures may only offer an artifactual indication of TCR flexibility, influenced by crystallization conditions and crystal packing that is inconsistent with the theoretical potential of intrinsic TCR motions.

## Introduction

T-cells constitute our primary cellular defense against pathogenic challenge and play a major role in controlling neoplasms. The key molecular interface that enables T-cells to sense these threats is mediated by the clonally expressed T cell receptor (TCR) that classically distinguishes between self and foreign peptides. These peptides are derived from processed intra- and extra-cellular proteins, presented by highly diverse major histocompatibility complexes (pMHCs) on the surface of most nucleated cells (1). TCRs are required to respond to a vast number of potential foreign peptides that they have not encountered before, are unable to adapt to, and that can be presented by multiple MHCs (2, 3). More recently, it has been shown that the TCR can also recognize lipid antigens and metabolites presented by the invariant MHC-like cluster of differentiation 1d and MHC class I-related molecules, respectively (4, 5). Further evidence has implicated several other MHC-like molecules as antigenic targets for T-cells, exemplifying the extreme versatility of the TCR (6, 7).

In order to tackle this vast antigenic milieu, the gene rearrangement process that produces the TCR provides almost limitless possible TCR sequences through the recombination of TCR variable, joining and diversity genes (the germline encoded component), as well as addition and deletion of nucleotides (the somatic component). Additionally, through the combination of two chains (α and β) to form a heterodimeric αβ TCR, it is theoretically possible to generate ~10^18^ TCRs in humans. However, only a small fraction (<10^8^) of these possibilities are ever expressed in any individual due to space limitations, suggesting that TCRs must be able to recognize multiple pMHCs to cover all potential pathogen encounters (8). Indeed, recent experimental evidence has confirmed this notion, demonstrating that TCRs can recognize millions of pMHCs with physiologically relevant sensitivity (9–13).

One key feature that is likely to facilitate this level of cross-reactivity is flexibility within the TCR complementarity-determining region loops (CDR loops) that form the antigen contact zone. Indeed, early thermodynamic evidence (14–16), combined with NMR spectroscopy (17–19) and fluorescence anisotropy (18, 20–22) has been used to indirectly and directly demonstrate TCR CDR loop motions. Alongside biophysical approaches, crystal structures comparing unbound and pMHC-bound TCRs have demonstrated that the TCR CDR loops can change shape, becoming stabilized upon binding (20, 23, 24). However, although X-ray crystallography provides unparalleled resolution of proteins too small for cryo-EM, the resulting snapshots represent only one conformational state that could be influenced by crystallographic artifacts. In order to extend the reach of atomic structures, computational modeling of protein motion has been used for almost 40 years to study a range of different systems ranging from enzymes, viral proteins, G protein coupled receptors and, more recently, immune receptors (21, 25–33). Application of this approach is beginning to shed light on the malleability of the TCR during pMHC binding, although questions still remain about the intrinsic flexibility of the TCR.

Here, we focused on two important unresolved questions. First, are conformational changes between unbound and pMHC-bound TCRs biologically relevant or crystal artifacts (especially considering that the CDR loops of unbound TCRs are more “exposed” for non-biologically relevant crystal contacts)? We addressed this issue by solving the structures of three different TCRs from multiple crystals. These included 12 datasets of the F11 TCR that recognizes a peptide from the influenza hemagglutinin protein (PKYVKQNTLKLAT) presented by HLA-DR*0101 (DR1-PKY), five data sets from the HA1.7 TCR that also recognizes DR1-PKY, and five datasets from the 003 TCR that recognizes an HIV p17 Gag-derived peptide (SLYNTVATL) presented by HLA-A*0201. These structures were compared to determine whether different loop conformations existed independent of ligand binding. Second, we investigated the intrinsic flexibility of the TCR CDR loops in unbound state by implementing geometric simulations of flexible TCR motion using a combination of rigidity analysis and coarse-grained elastic network normal mode analysis.

Overall, our data support the notion that crystal structures may represent an artifactual indication of TCR CDR loop flexibility that offers only a snapshot of the theoretical potential of TCR CDR loop motions. These data provide additional evidence contributing toward our understanding of the molecular mechanisms that mediate T-cell antigen discrimination and cross-reactivity.

## Materials and Methods

### Protein Expression, Refolding, and Purification

The F11, HA1.7, and 003 TCRs were generated as previously described (34), and the α and β chains were cloned into separate pGMT7 expression plasmids under the control of the T7 promoter. Each TCR was refolded and purified using methods that have been described previously (35).

### Crystal Structure Determination

All protein crystals were grown at 18°C by vapor diffusion *via* the sitting drop technique. 200 nL of each TCR (10 mg/ml) in crystallization buffer (10 mM Tris pH 8.1 and 10 mM NaCl) was added to 200 nL of reservoir solution. The TCR crystals used in the structural investigations were grown in a variety of different conditions from PACT premier™ HT-96, JBScreen Classic HTS I, or TOPS (36) detailed in Table 1. Crystallization screens were conducted using an Art-Robbins Phoenix dispensing robot (Alpha Biotech Ltd., UK) and data were collected at 100 K at the diamond light source (DLS), Oxfordshire, UK using an ADSC Q315 CCD detector. Reflection intensities were estimated using XIA2 (37) and the data were analyzed with SCALA and the CCP4 package (38). Structures were solved with molecular replacement using PHASER (39). Sequences were adjusted with COOT (40) and the models were refined with REFMAC5. Graphical representations were prepared with PYMOL (41). Crystal contacts were determined using PYMOL and defined as intermolecular distances <4.0 Å. The reflection data and final model coordinates were deposited in the PDB database and are detailed in Tables 2–4.

**Table 1:** Crystallization conditions for TCR structures.

Crystal	Crystal growth conditions
F11 003	18% PEG 4 K, 100 mM sodium acetate pH 4.5
F11 011	20% PEG 4 K, 200 mM ammonium sulfate
F11 034	25% PEG 1.5 K, 100 mM SPG pH 6.0
F11 036	25% PEG 1.5 K, 100 mM SPG pH 5.0
F11 041	25% PEG 1.5 K, 100 mM MMT pH 6.0
F11 046	20% PEG 3.35 K, 200 mM sodium fluoride
F11 053	20% PEG 3.35 K, 20 mM sodium phosphate
F11 054	20% PEG 3.35 K, 200 mM sodium malonate
F11 055	20% PEG 3.35 K, 200 mM sodium acetate trihydrate
F11 058	20% PEG 3.35 K, 200 mM sodium sulfate
F11 061	20% PEG 3.35 K, 200 mM potassium thiocyanate

F11 081	25% PEG 4 K, 200 mM ammonium sulfate, 100 mM MES pH 7.0
HA1.7 010	20% PEG 3.35 K, 200 mM potassium thiocyanate
HA1.7 049	20% PEG 3.35 K, 200 mM potassium thiocyanate
HA1.7 054	20% PEG 3.35 K, 200 mM potassium thiocyanate
HA1.7 077	15% PEG 4 K, 15% glycerol, 100 mM MES pH 7.0
HA1.7 079	15% PEG 4 K, 15% glycerol, 100 mM MES pH 7.0

003 007	25% PEG 4 K, 200 mM ammonium sulfate, 100 mM HEPES pH 7.0
003 035	15% PEG 4 K, 15% glycerol, 100 mM TRIS pH 7.5
003 037	25% PEG 4 K, 15% glycerol, 100 mM TRIS pH 8.0
003 041	20% PEG 4 K, 200 mM ammonium sulfate, 100 mM sodium cacodylate pH 6.0
003 042	20% PEG 4 K, 200 mM ammonium sulfate, 100 mM sodium cacodylate pH 6.5

*All above conditions are from PACT premier™ HT-96, JBScreen Classic HTS I, or TOPS (36)*.

**Table 2:** Data collection and refinement statistics for F11 TCR structures.

PDB code	6FR9	6FRA	6EH7	6FRB
**Data collection statistics**				
Diamond beamline	DLS I04-1	DLS I04-1	DLS I04-1	DLS I04-1
Space group	P 21 21 2	P 21 21 2	P 21 21 2	P 21 21 2
Wavelength (Å)	0.92	0.92	0.92	0.92
Crystal number	003	011	034	036
**Cell dimensions**				
*a, b, c* (Å)	85.5, 115.4, 50.9	85.7, 114.6, 50.7	85.4, 114.5, 50.6	85.2, 115.4, 50.5
α, β, γ (°)	90.0, 90.0, 90.0	90.0, 90.0, 90.0	90.0, 90.0, 90.0	90.0, 90.0, 90.0
Resolution (Å)	1.62–47.81	1.73–47.64	1.89–46.30	1.75–47.78
Outer shell	1.62–1.66	1.73–1.78	1.89–1.94	1.75–1.80
*R*_merge_ (%)	4.9 (76.8)	6.0 (78.3)	5.6 (70.6)	5.8 (116.1)
*R*_meas_ (%)	5.3 (83.3)	6.5 (84.7)	6.1 (76.5)	6.4 (125.8)
CC1/2	0.999 (0.801)	0.999 (0.826)	0.999 (0.852)	0.999 (0.771)
*I*/σ*I*	20.6 (2.7)	19.2 (2.8)	22.3 (2.8)	18.5 (1.7)
Completeness (%)	99.9 (100)	99.9 (100)	98.7 (98.7)	99.9 (99.9)
Redundancy	6.6 (7.0)	6.6 (6.9)	6.5 (6.8)	6.5 (6.8)
Unique reflections	64,727 (4,734)	52,808 (3,853)	39,943 (2,917)	50,657 (3,716)
**Refinement statistics**				
*R*-work reflections	61,390	50,066	37,906	48,036
*R*-free reflections	3,280	2,690	2,005	2,569
*R*_work_/*R*_free_	17.9/21.1	20.3/24.3	18.5/23.2	21.0/24.3
**R.m.s. deviations**				
Bond lengths (Å)	0.017	0.019	0.019	0.020
Bond angles (°)	1.902	2.038	1.926	2.086
Coordinate error^a^	0.064	0.082	0.103	0.095
Mean *B* value (Å^2^)	26.9	29.8	33.9	32.1
**Ramachandran statistics**				
Favoured/allowed/outliers	390/14/1	408/13/3	419/19/0	408/19/2
(%)	96.3/3.5/0.2	95.1/4.2/0.7	96/4/0	95.1/4.4/0.5

**PDB code**	**6EH6**	**6FRC**	**6FUM**	**6FUN**

**Data collection statistics**				
Diamond beamline	DLS I04-1	DLS I04-1	DLS I04-1	DLS I04-1
Space group	P 21 21 2	P 21 21 2	P 21 21 2	P 21 21 2
Wavelength (Å)	0.92	0.92	0.92	0.92
Crystal number	041	046	053	054
**Cell dimensions**				
*a, b, c* (Å)	85.8, 114.1, 50.7	85.3, 114.8, 50.7	85.6, 114.2, 50.6	85.1, 115.3, 50.7
α, β, γ (°)	90.0, 90.0, 90.0	90.0, 90.0, 90.0	90.0, 90.0, 90.0	90.0, 90.0, 90.0
Resolution (Å)	1.78–50.72	1.59–46.38	1.76–46.28	1.58–46.37
Outer shell	1.78–1.83	1.59–1.63	1.76–1.81	1.58–1.62
*R*_merge_ (%)	4.5 (73.7)	4.3 (72.0)	5.8 (69.4)	4.1 (65.4)
*R*_meas_ (%)	5.3 (87.5)	4.7 (77.9)	6.3 (75.2)	4.5 (70.7)
CC1/2	1.000 (0.818)	1.000 (0.824)	0.999 (0.854)	0.999 (0.841)
*I*/σ*I*	22.5 (2.4)	22.7 (2.9)	17.4 (2.6)	23.6 (3.1)
Completeness (%)	99.9 (100.0)	99.7 (100)	99.7 (99.9)	99.0 (100)
Redundancy	6.5 (6.8)	6.6 (6.9)	6.4 (6.8)	6.6 (7.0)
Unique reflections	48,509 (3,524)	67,125 (4,908)	49,905 (3,658)	69,019 (5,006)
**Refinement statistics**				
*R*-work reflections	46,009	63,669	47,326	65,474
*R*-free reflections	2,450	3,398	2,530	3,488
*R*_work_/*R*_free_	19.4/22.4	17.7/20.6	18.1/22.3	17.4/20.7
**R.m.s. deviations**				
Bond lengths (Å)	0.020	0.018	0.018	0.017
Bond angles (°)	2.008	1.952	1.880	1.904
Coordinate error^a^	0.094	0.058	0.087	0.055
Mean *B* value (Å^2^)	33.0	27.2	31.2	26.7
**Ramachandran statistics**				
Favoured/allowed/outliers	421/17/0	377/16/1	399/19/1	382/17/1
(%)	96/4/0	95.7/4.1/0.2	95.2/4.5/0.2	95.5/4.3/0.2

**PDB code**	**6FUO**	**6FUP**	**6FUQ**	**6FUR**

**Data collection statistics**				
Diamond beamline	DLS I04-1	DLS I04-1	DLS I04-1	DLS I04-1
Space group	P 21 21 2	P 21 21 2	P 21 21 2	P 21 21 1
Wavelength (Å)	0.92	0.92	0.92	0.92
Crystal number	055	058	061	081
**Cell dimensions**				
*a, b, c* (Å)	85.4, 115.2, 50.8	85.3, 115.2, 50.7	85.4, 114.8, 50.7	50.7, 114.9, 85.3
α, β, γ (°)	90.0, 90.0, 90.0	90.0, 90.0, 90.0	90.0, 90.0, 90.0	90.0, 91.1, 90.0
Resolution (Å)	1.70–46.47	1.72–46.41	1.60–50.73	1.73–46.34
Outer shell	1.70–1.74	1.72–1.77	1.60–1.65	1.73–1.77
*R*_merge_ (%)	4.5 (62.7)	4.1 (74.1)	4.6 (72.9)	7.4 (65.6)
*R*_meas_ (%)	4.9 (67.8)	4.5 (80.2)	5.0 (78.7)	8.8 (77.7)
CC1/2	0.999 (0.867)	1.000 (0.814)	0.999 (0.827)	0.996 (0.663)
*I*/σ*I*	22.7 (3.2)	23.6 (2.7)	19.8 (2.7)	11.1 (2.0)
Completeness (%)	99.1 (99.7)	98.6 (99.4)	99.7 (99.8)	94.8 (98.6)
Redundancy	6.6 (6.9)	6.6 (6.9)	6.6 (7.0)	3.3 (3.5)
Unique reflections	55,910 (4,094)	53,476 (3,936)	65,936 (4,828)	96,229 (7,382)
**Refinement statistics**				
*R*-work reflections	53,021	50,709	62,540	91,379
*R*-free reflections	2,835	2,715	3,342	4,819
*R*_work_/*R*_free_	16.8/20.0	17.6/21.5	17.3/21.2	18.9/22.6
**R.m.s. deviations**				
Bond lengths (Å)	0.018	0.016	0.016	0.018
Bond angles (°)	1.922	1.847	1.862	1.901
Coordinate error^a^	0.069	0.075	0.061	0.092
Mean *B* value (Å^2^)	29.6	33.5	29.5	26.4
**Ramachandran statistics**				
Favoured/allowed/outliers	388/14/1	396/18/1	392/14/1	800/34/2
(%)	96.3/3.5/0.2	95.4/4.3/0.2	96.3/3.4/0.3	95.7/4.1/0.2

*One crystal was used for determining each structure*.

*Figures in brackets refer to outer resolution shell, where applicable*.

*^a^Coordinate estimated standard uncertainty in (Å), calculated based on maximum likelihood statistics*.

**Table 3:** Data collection and refinement statistics for HA1.7 T cell receptor structures.

PDB code	6FR6	6FR7	6FR8	6EH8	6EH9
**Data collection**					
Diamond beamline	DLS I02	DLS I03	DLS I02	DLS I04-1	DLS I04-1
Space group	P1 21 1	P1 21 1	P1 21 1	P1 21 1	P1 21 1
Wavelength (Å)	0.98	0.98	0.98	0.92	0.92
Crystal number	010	049	054	077	079
**Cell dimensions**					
*a, b, c* (Å)	70.0, 50.2, 73.2	69.4, 50.0, 72.8	69.5, 49.9, 72.9	69.2, 49.5, 72.6	69.5, 50.0, 72.8
α, β, γ (°)	90.0, 93.3, 90.0	90.0, 94.5, 90.0	90.0, 94.3, 90.0	90.0, 94.7, 90.0	90.0, 93.1, 90.0
Resolution (Å)	2.98–36.52	2.31–72.53	2.38–69.34	2.51–52.08	2.49–48.87
Outer shell	2.98–3.06	2.31–2.37	2.38–2.44	2.51–2.58	2.49–2.55
*R*_merge_ (%)	9.60 (54.0)	7.7 (86.2)	6.5 (110.1)	11.6 (138.8)	4.3 (52.0)
*R_meas_* (%)	13.2 (73.6)	10.8 (107.7)	9.8 (163.8)	14.7 (170.6)	6.0 (70.3)
CC1/2	N/A	N/A	N/A	N/A	N/A
*I/*σ*I*	11.6 (2.3)	9.2 (2.5)	9.9 (2.1)	12.4 (2.3)	17.5 (2.3) (99.2)
Completeness (%)	98.8 (99.5)	97.1 (97.3)	98.8 (99.6)	98.2 (97.9)	98.5 (99.2)
Redundancy	4.0 (4.1)	3.7 (3.9)	3.5 (3.7)	3.8 (3.9)	3.7 (3.9)
Unique reflections	10,468 (782)	21,302 (1,562)	19,994 (1,444)	16,674 (1,196)	17,434 (1,273)
**Refinement**					
R-work reflections	9,958	20,190	18,937	15,735	16,545
R-free reflections	736	1,094	1,019S	837	881
R_work_/R_free_	20.1/29.9	21.9/27.7	22.2/27.8	22.6/29.6	23.2/29.7
**R.m.s. deviations**					
Bond lengths (Å)	0.016	0.018	0.017	0.011	0.013
Bond angles (°)	1.957	1.938	1.939	1.55	1.65
Coordinate error^a^	0.45	0.25	0.29	0.32	0.30
Mean B value (Å^2^)	57.7	58.7	67.1	63.3	65.7
**Ramachandran statistics**					
Favored/allowed/outliers	399/29/7	407/24/2	409/21/2	409/28/3	409/23/9
(%)	91.7/6.7/1.6	94.0/5.5/0.5	94.7/4.9/0.5	93/6/1	93/5/2

*One crystal was used for determining each structure*.

*Figures in brackets refer to outer resolution shell*.

*^a^Coordinate estimated standard uncertainty in (Å), calculated based on maximum likelihood statistics*.

**Table 4:** Data collection and refinement statistics for 003 T cell receptor structures.

PDB code	6FR3	6FR4	6EH4	6FR5	6EH5
**Data collection**					
Diamond light source (DLS) beamline	DLS I04-1	DLS I04-1	DLS I04-1	DLS I04-1	DLS I04-1
Space group	P1 21 1	P1 21 1	P1 21 1	P1 21 1	P1 21 1
Wavelength (Å)	0.92	0.92	0.92	0.92	0.92
Crystal number	007	035	037	041	042
**Cell dimensions**					
*a, b, c* (Å)	43.1, 81.4, 64.8	43.3, 81.3, 65.1	43.2, 81.2, 65.1	43.1, 81.2, 64.8	43.2, 81.2, 64.9
α, β, γ (°)	90.0, 90.1, 90.0	90.0, 90.3, 90.0	90.0, 90.3, 90.0	90.0, 90.4, 90.0	90.0, 90.3, 90.0
Resolution (Å)	1.35–50.69	1.28–43.28	1.26–43.23	1.37–40.59	1.29–50.69
Outer shell	1.45–1.39	1.28–1.31	1.26–1.29	1.37–1.41	1.29–1.32
*R*_merge_ (%)	4.1 (49.6)	4.2 (42.3)	3.6	4.9 (54.5)	4.1 (43.1)
*R_meas_* (%)	5.6 (58.7)	4.9 (54.3)	5.1 (62.1)	5.7 (63.8)	4.8 (53.5)
CC1/2	0.998 (0.752)	0.996 (0.725)	0.998 (0.674)	0.998 (0.736)	0.999 (0.734)
*I/*σ*I*	13.9 (2.3)	15.5 (2.2)	15.5 (2.2)	13.7 (2.3)	16.1 (2.4)
Completeness (%)	97.8 (92.5)	94.8 (67.1)	87.9 (45.8)	98.4 (95.8)	94.9 (65.3)
Redundancy	3.8 (3.5)	3.6 (2.6)	3.8 (3.2)	3.8 (3.7)	3.7 (2.9)
Unique reflections	95,658 (6,641)	109,519 (5,708)	106,284 (4,045)	91,903 (6,599)	106,513 (5,427)
**Refinement**					
R-work reflections	90,837	104,016	100,974	87,272	101,171
R-free reflections	4,793	5,421	5,303	6,273	5,313
R_work_/R_free_	16.5/19.7	16.8/19.0	15.8/19.1	16.8/19.1	17.0/19.5
**R.m.s. deviations**					
Bond lengths (Å)	0.023	0.019	0.023	0.017	0.035
Bond angles (°)	2.341	1.977	2.22	1.842	2.69
Coordinate error^a^	0.04	0.04	0.04	0.04	0.04
Mean B value (Å^2^)	21.0	17.8	18.7	19.2	18.9
**Ramachandran statistics**					
Favored/allowed /outliers	398/11/0	339/11/0	459/8/2	372/10/0	461/8/0
(%)	97.3/2.7/0	96.9/3.4/0	98/2/0	97.4/2.6/0	98/2/0

*One crystal was used for determining each structure*.

*Figures in brackets refer to outer resolution shell*.

*^a^Coordinate estimated standard uncertainty error calculated based on maximum likelihood statistics*.

### Geometric Simulations of Flexible Motion

Amplitudes of motion in representative structures of the unbound TCRs solved here were simulated using a combination of rigidity analysis and coarse-grained elastic network normal mode analysis. Elnemo software (42) was used to obtain normal mode eigenvectors from coarse-grained elastic network modeling. FIRST/FRODA software (43, 44) was used to carry out rigidity analysis (FIRST) (45), which identified the noncovalent interaction network and labeling dihedral angles as locked or variable, and template-based geometric simulations of flexible motion (FRODA) (44) which project the all-atom structure over large amplitudes of motion, while maintaining local bonding and steric geometry.

Normal mode eigenvectors were generated in Elnemo in a one-site-per-residue coarse-graining using the Cα geometry of the input structure, placing springs of equal spring constant between all sites lying within an interaction distance cut-off of 12 Å. A rigidity analysis of the all-atom input structure was carried out in FIRST using the “pebble game” algorithm (43, 46), which matches degrees of freedom against bonding constraints in the molecular framework of the protein. Bonding constraints, include covalent, hydrophobic, and polar (hydrogen bond and salt bridge) interactions. As the strength of the polar interactions can be gauged from their geometry, the results of the analysis depend on an “energy cut-off” which selects the set of polar interactions to include in the constraint network (45). A cut-off of −3.0 kcal/mol was used in this study for simulations of flexible motion. We explored flexible motion biased along the 10 lowest-frequency nontrivial normal modes identified by Elnemo (modes 7–16; modes 1–6 are trivial rigid body motions).

Template-based geometric simulation of flexible motion, carried out using FRODA, explores the mobility of the all-atom structure by iterative perturbation and relaxation of atomic positions in parallel and antiparallel to the direction of normal mode eigenvectors. Several thousand iteration steps were carried out to generate large motion amplitudes. The simulation generates an initial phase of “easy” motion, where the bonding geometry is easily maintained, followed by the onset of “jamming” as the motion encounters steric and bonding constraints, which naturally limit its amplitude. The conformational changes of geometric simulations of TCRs projected using this method were observed, and compared with superpositions of crystallographic models in PyMOL.

## Results

### TCR CDR Loop Flexibility Analysis Using X-Ray Crystallography

Several investigators, including ourselves, have previously used the structures of unbound and pMHC-bound TCRs to explore conformational changes that occur during pMHC ligand binding (20, 23, 24, 35). These studies have revealed that some TCRs undergo large conformational changes during binding, whereas others use a “lock-and-key”-type ligation strategy. However, questions remain over whether these changes accurately reflect how TCRs engage pMHC, or whether these observations are biased because they rely on a static image of a highly flexible protein interface that could be further affected by crystal lattice contacts, crystal packing, and/or crystallization conditions. To address this question, we solved multiple structures of three unbound TCRs at atomic resolution. We generated 12 structures of the F11 TCR, that recognizes a peptide from the influenza hemagglutinin protein (PKYVKQNTLKLAT) presented by HLA-DR*0101 (DR1-PKY), between 1.58 and 1.89 Å resolutions; 5 structures of the DR1-PKY specific HA1.7 TCR, between 2.31 and 2.98 Å resolution; and five structures of the 003 TCR, that recognizes an HIV-GAG-derived peptide (SLYNTVATL) presented by HLA-A*0201 (A2-SLY), between 1.26 and 1.37 Å resolution. All of the structures were solved with crystallographic R_work_/R_free_ ratios within accepted limits as shown by the theoretically expected distribution (47). Statistical analysis and structure factors from two representative structures from each TCR are shown in Tables 2–4. The structures were refined by multiple individuals to avoid bias during refinements.

**Figure 1:**
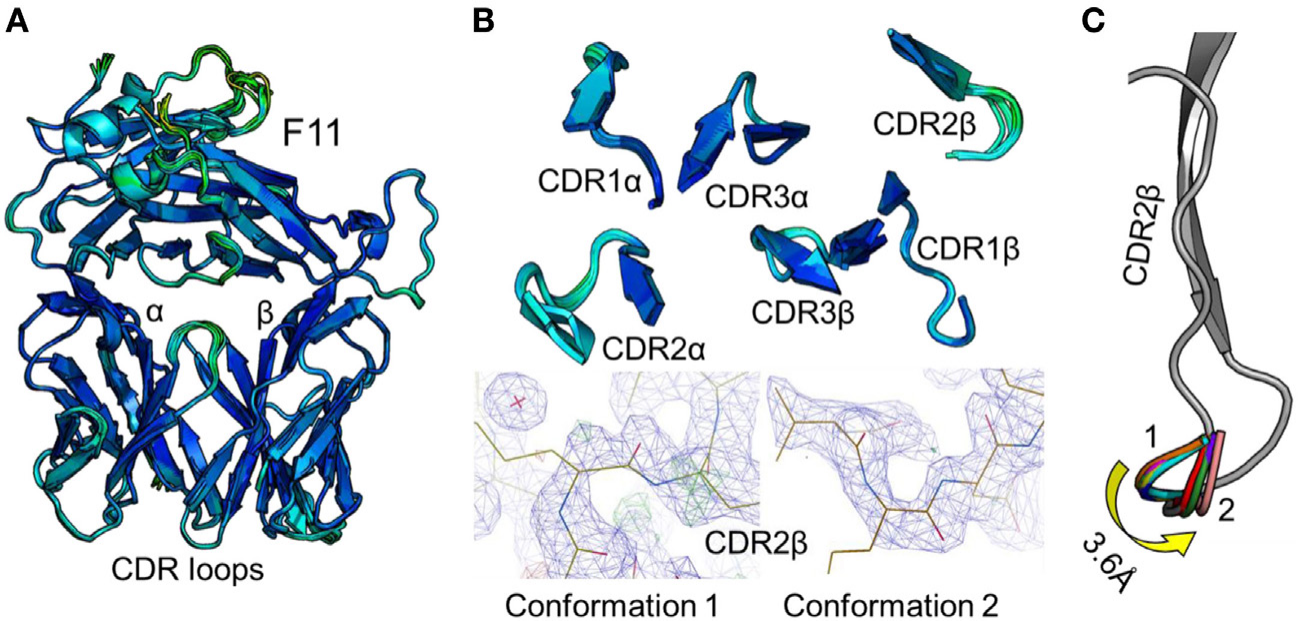
Comparison of 12 unbound structures of the F11 T cell receptor (TCR). **(A)** Side-on view of the overall conformation of the F11 TCR (cartoon, colored by B-factor) including alignment of structures generated from 12 different crystals. **(B)** Top down view of an alignment of the complementarity determining region (CDR) loops (cartoon, colored by B-factor) from the 12 structures of unbound F11 TCR. The panels below demonstrate the two extreme conformations (conformation 1 and 2) of the CDR2β loop from the different structures, with the observed electron density map at 1σ. **(C)** Side-on view of the F11 TCR CDR2β loop (framework region in gray, apex of the loop in multiple colors) aligning all 12 structures with conformation 1 and conformation 2 labeled.

**Figure 2:**
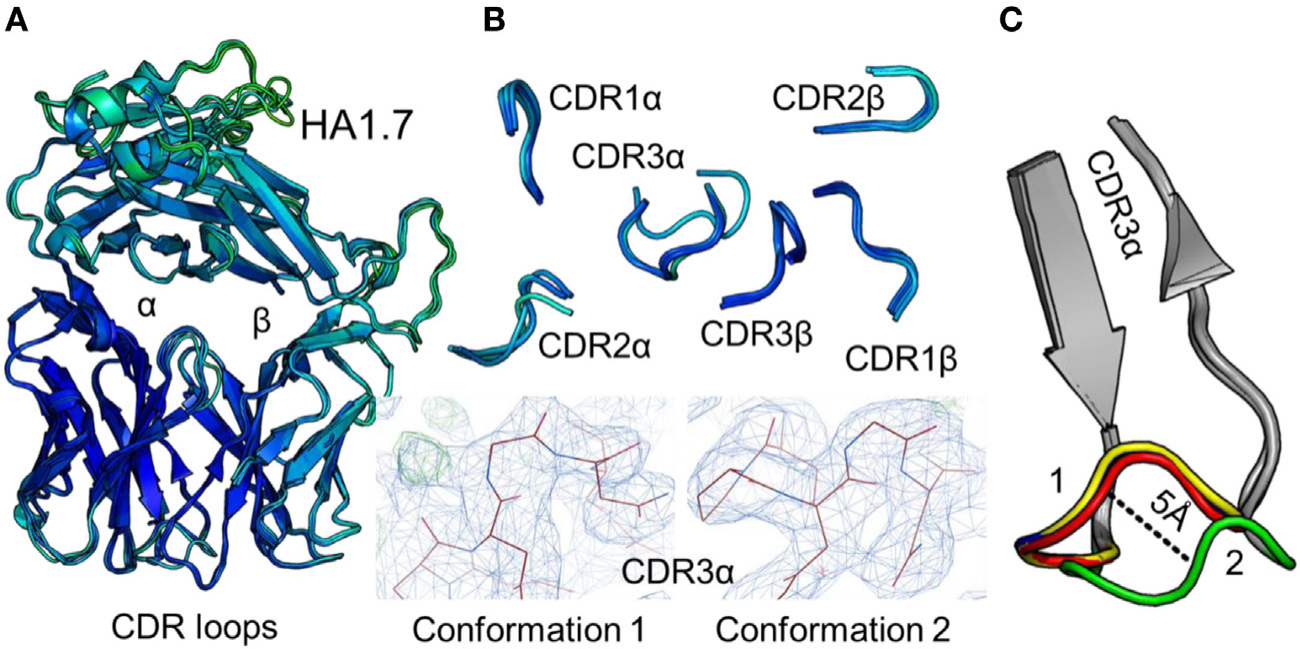
Comparison of five unbound structures of the HA1.7 T cell receptor (TCR). **(A)** Side-on view of the overall conformation of the HA1.7 TCR (cartoon, colored by B-factor) including alignment of structures generated from five different crystals. **(B)** Top down view of an alignment of the complementarity determining region (CDR) loops (cartoon, colored by B-factor) from the five structures of unbound HA1.7 TCR. The panels below demonstrate the two extreme conformations (conformation 1 and 2) of the CDR3α loop from the different structures, with the observed electron density map at 1σ. **(C)** Side-on view of the HA1.7 TCR CDR3α loop (framework region in gray, apex of the loop in multiple colors) aligning all five structures with conformation 1 and conformation 2 labeled.

**Figure 3:**
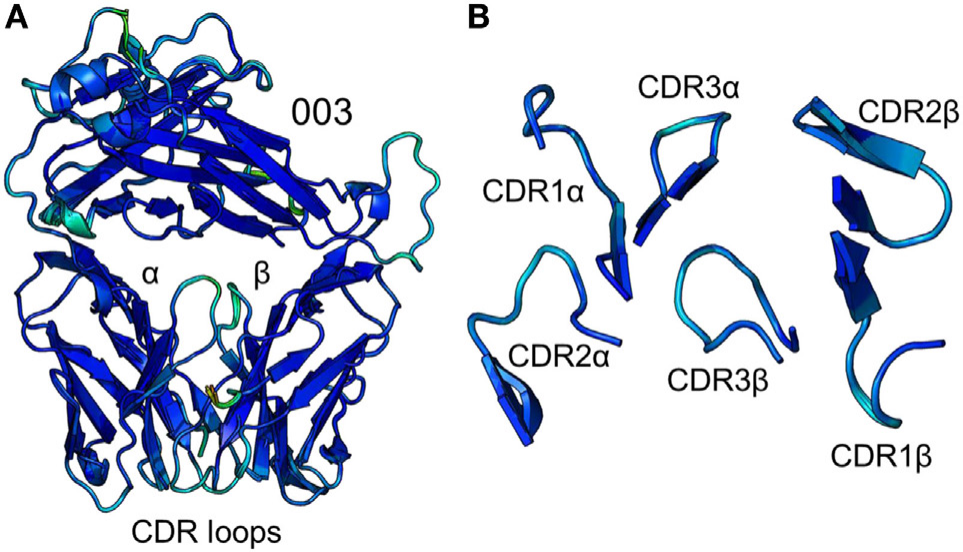
Comparison of five unbound structures of the 003 T cell receptor (TCR). **(A)** Side-on view of the overall conformation of the 003 TCR (cartoon, colored by B-factor) including alignment of structures generated from five different crystals. **(B)** Top down view of an alignment of the complementarity determining region loops (cartoon, colored by B-factor) from the five structures of unbound 003 TCR.

To accurately investigate TCR CDR loop movement during pMHC binding using crystal structures, the unbound TCR structures should, ideally, all be identical. We found that this was not the case for two of the three TCRs under investigation. Indeed, for the F11 TCR, we observed Cα backbone flexibility in the CDR2β loop, shifting by up to 3.6 Å in different structures of the same protein (Figure 1). For the HA1.7 TCR, we observed a larger shift in potential positions for the Cα backbone of the CDR3α loop, differing by up to 5 Å (Figure 2). In both cases, these shifts were comparable to loop movements reported in several other studies in which the unbound and pMHC-bound TCRs were compared (23). These findings demonstrate the potential for artifactual interpretation of the mechanism of TCR ligation using structures alone. However, this is not always the case. Indeed, the CDR loops of the third TCR included in our study, the 003 TCR, were superimposable in all the structures solved (Figure 3). B-factor analysis did not correlate with these loop movements (Figures 1–3).

Having observed positional differences in CDR loop positions in multiple, but not all, TCR datasets, we hypothesized that such differences might be explained by crystallographic artifacts. We therefore considered whether the resolution of the structures, or the crystal lattice contacts, had an impact on the nature of the loop movements observed in our structures. The 003 TCR structures, which exhibited practically identical CDR coordinates, were solved at an average resolution of 1.31 Å. In contrast, the HA1.7 TCR structures, in which a large loop movement was observed, were solved at 2.5 Å average resolution. This difference in resolution could partly explain why the interpretation of the position of the CDR3α loop of the HA.17 TCR varied between structures if the density was poor in this region. However, there was clear density supporting both conformations in the HA1.7 TCR structures (Figure 2B). Moreover, we also observed loop movement for the F11 TCR structures, which were solved at an average resolution of 1.55 Å, comparable to the higher resolution datasets for the 003 TCR. Last, we investigated whether stabilizing crystal lattice contacts might explain why some of the CDR loops appeared identical while others could shift between structures. For the F11 TCR, only the CDR2α and CDR2β loops were free from any lattice contacts (data not shown). Thus, the ability of the CDR2β loop to shift between structures could have been partly due to extra freedom imparted by individual crystal packing. Similarly, the HA1.7 TCR CDR3α loop was free from crystal lattice contacts and shifted between structures. However, several other loops in both the F11 and HA1.7 TCR structures were also free from crystal lattice contacts and did not shift between structures. Finally, none of the 003 TCR CDR loops, which were identical in each structure, made any potentially stabilizing crystal lattice contacts. Overall, neither the resolution of the structures, or the availability of stabilizing crystal lattice contacts were good predictors of TCR CDR loop shifts between structures. Thus, we conclude that CDR loop movements observed between unbound and pMHC-bound TCRs require further investigation in order to confirm whether they are artifactual, or a real part of the TCR-binding mechanism.

### Investigating TCR CDR Loop Flexibility Using Rigidity Analysis and Simulations of Protein Motion

Direct measurements of protein flexibility at the single loop level is highly challenging because of the size (nm) and time (ms) of the movements. Indirect measurements using individually labeled amino acids are possible using NMR and other techniques, but these experiments are technically challenging, time consuming, and not universally available. As an alternative approach, computational modeling has developed rapidly over the past few years and has emerged as a useful technique to investigate protein motions (31). However, for T-cell recognition studies, most of these modeling approaches have focused on flexibility at the peptide-MHC, or TCR-pMHC interface rather than exploring the motional potential of the TCR in unbound state. In the one study that did use this method to investigate TCR flexibility, the authors found large differences in flexibility between two different TCRs that helped to explain the antigen-recognition mechanisms employed by each TCR, demonstrating the usefulness of this approach (21).

Here, we investigated the intrinsic rigidity and flexibility of the unbound TCR structure datasets using pebble-game rigidity analysis, elastic network modeling, and geometric simulations of flexible motion, using a combination of Elnemo and FIRST/FRODA software (44). FIRST software identifies the network of noncovalent constraints in the system, including both polar (hydrogen bond) and hydrophobic-tether interactions. Polar interactions are assigned strength in the range 0 to −10 kcal/mol based on their geometry. The set of polar interactions to include in the rigidity analysis is controlled by an energy cutoff parameter E_cut_. We have demonstrated that biologically significant flexibility can be explored at cutoffs in the range −2 to −4 kcal/mol (48, 49). In order to determine the appropriate E_cut_ for TCRs, we performed rigidity analysis using the 003 TCR at cutoffs of −2 kcal/mol and −3 kcal/mol (data not shown). Analysis at a cutoff of −3 kcal/mol, but not −2 kcal/mol, demonstrated that the structure was largely flexible, with very few large rigid clusters. However, N and C terminal domains were still rich in noncovalent interactions maintaining the secondary, tertiary, and quaternary structure. We, therefore, explored flexible motion in all three TCR structures using the constraint network found at E_cut_ = −3 kcal/mol.

**Figure 4:**
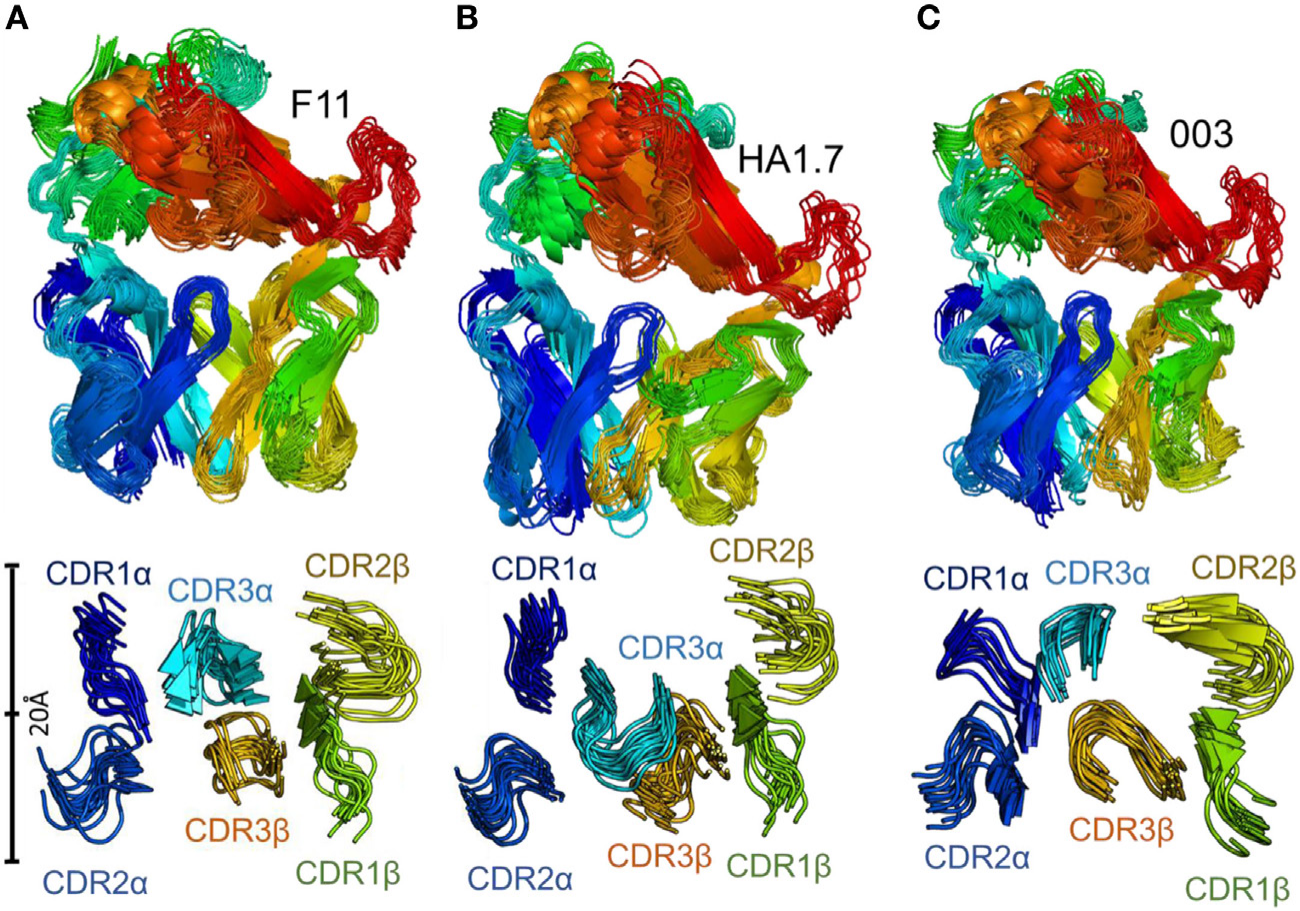
Flexibility analysis of the unbound F11, HA1.7, and 003 T cell receptor (TCRs). The TCRs were subjected to analysis using the FRODA/FIRST software packages to investigate protein flexibility. The top panels show the overall conformation of each TCR, using a single dataset, in which the TCR could flex over up to 2,000 frames, or until the run stopped because of steric clashes or overextended constraints. The bottom panel displays only the CDR loops of each TCR, with a distance scale to measure the motions, using the same analysis. **(A)** F11 TCR. **(B)** HA1.7 TCR. **(C)** 003 TCR. All structures are colored by protein domain, with the CDR1α in dark blue, CDR2α in blue, CDR3α in cyan, CDR1β in green, CDR2β in yellow, and CDR3β in orange.

We used Elnemo software to identify the 10 lowest-frequency nontrivial normal modes in each TCR structure. We then used the FRODA module of FIRST to project the structure along each mode, while retaining the local covalent and noncovalent bonding geometry of the input structure. This represents intrinsic flexible motion of the structure which can easily be explored in solution. Recent work has shown that the character of motion identified using this method is consistent with conventional molecular dynamics (MD) simulations, while requiring minimal computational expense (a few CPU-hours) (49, 50). The lowest-frequency modes include substantial components of relative domain motions, in which the interdomain section of each chain (around residues 110–120) provides a flexible joint, as was also recently observed in the large dimeric enzyme Dcps (49). As a result, a structural overlay of conformations generated by FRODA (Figure 4) includes both domain-motion variations and local changes in loop geometry.

**Figure 5:**
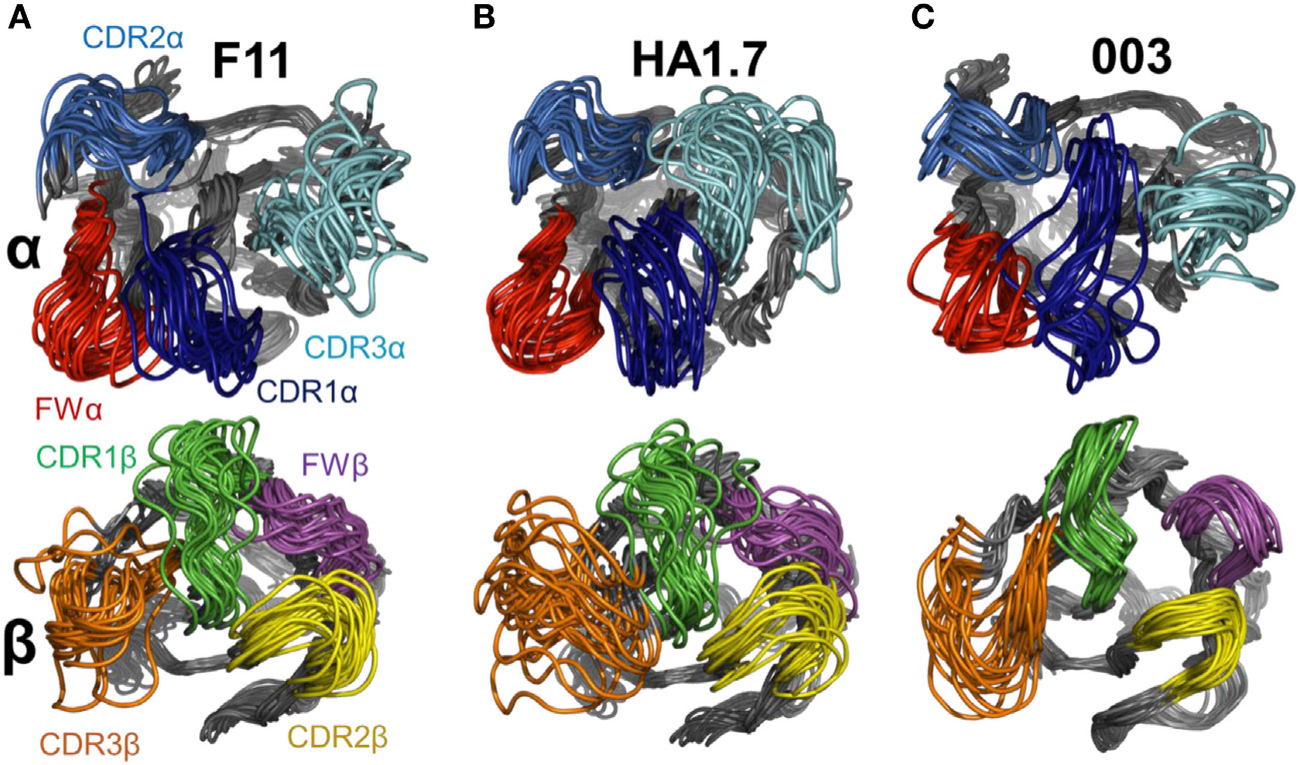
Flexible variations in the T cell receptor (TCR) complementarity determining region (CDR) loops using FRODA simulations. To isolate the loop motions, an alignment on the *N*-terminal domain for each TCR was performed (residues 6–110) to allow visualization of the loop structural variations relative to a stable base of comparison. Each panel shows a set of 20 structural variants aligned onto the initial crystal structure. Top panel TCRα CDR loops, bottom panel TCRβ CDR loops. For each normal mode, we selected the variants representing the natural limit of flexible motion parallel and antiparallel to the mode direction. The four loops are colored as follows: CDR1α in dark blue, CDR2α in blue, CDR3α in cyan, FWα in red, CDR1β in green, CDR2β in yellow, CDR3β in orange, and FWβ in purple. **(A)** F11 TCR. **(B)** HA1.7 TCR. **(C)** 003 TCR.

To isolate the loop motions specifically, we carried out an alignment on the *N*-terminal domain for each structure and each chain (residues 6–110). The alignment was carried out in PyMOL on the non-loop residues of each domain (Table 5). This allowed visualization of CDR loop structural variations relative to a stable base of comparison, a set of 20 generated structural variants (Figure 5). For each normal mode, we selected the variants representing the natural limit of flexible motion parallel and antiparallel to the mode direction. This natural limit is the point at which covalent and noncovalent constraints (including steric contacts) start to limit the amplitude of the motion, such that further progress along the mode direction is “jammed”.

**Table 5:** Domain and loop identification in T cell receptor (TCRs).

TCR structure	F11 ($\bar{x}$ = 8.7)	HA1.7 ($\bar{x}$ = 9.0)	003 ($\bar{x}$ = 6.4)
**Loops in TCRα chain (amplitude at the apex of each loop in Å)**			
Complementarity determining region (CDR1)α ($\bar{x}$ = 7.2)	25–30 (7.7)	25–30 (8.0)	27–34 (6.0)
CDR2α ($\bar{x}$ = 7.7)	49–54 (9.4)	49–54 (6.9)	52–57 (6.8)
Fwα ($\bar{x}$ = 8.7)	66–72 (8.4)	66–72 (11.0)	68–74 (6.8)
CDR3α ($\bar{x}$ = 10.2)	93–98 (10.6)	93–101 (9.9)	95–100 (10.2)

**Loops in TCRβ chain (amplitude at the apex of each loop in Å)**			
CDR1β ($\bar{x}$ = 7.2)	23–29 (8.3)	26–31 (9.0)	27–32 (4.4)
CDR2β ($\bar{x}$ = 5.5)	48–53 (6.5)	49–54 (6.9)	50–55 (3.0)
Fwβ ($\bar{x}$ = 5.8)	66–72 (6.5)	69–74 (6.1)	69–74 (4.8)
CDR3β ($\bar{x}$ = 12.2)	92–98 (12.2)	96–102 (14.9)	95–102 (9.4)

Stable base region (TCRα)	6–110	6–110	6–110
Not loops	6–24, 31–48, 55–65, 73–92, 99–110	6–24, 31–48, 55–65, 73–92, 102–110	6–26, 35–51, 58–67, 75–94, 101–110

Stable base region (TCRβ)	6–110	6–110	6–110
Not loops	6–22, 30–47, 54–65, 73–91, 99–110	6–25, 32–48, 55–68, 75–95, 103–110	6–26, 33–49, 56–68, 75–94, 103–110

This analysis demonstrated that the scope for flexible variation in the loop geometries was substantial in all of the TCRs. Measurements of the maximal amplitude of the apex of each loop were conducted to provide estimations of the potential flexibility of each loop (Table 5). Although this analysis is an approximation and should be treated as such, the average maximal loop motion was slightly less (6.4 Å) for the 003 TCR compared to F11 (8.7 Å) and HA1.7 (9.0 Å), consistent with the structural data demonstrating greater rigidity in the 003 TCR. Further dissection of the data revealed that the longer somatically rearranged CDR3 loops had the most potential for loop motion (CDR3α: 10.2 Å, CDR3β: 12.2 Å) compared to the shorter germline encoded loops (CDR1α: 7.2 Å, CDR2α: 7.7 Å, Fwα: 8.7 Å, CDR1β:7.2 Å, CDR2β: 5.5 Å, Fwβ: 5.8 Å). These findings are consistent with the fact that the CDR3 loops generally make more interactions with the variable peptide component of the antigen, whereas the other loops are generally more focused toward the MHC surface. More generally, this analysis also indicated that all the structures, in solution, can explore large variations in loop geometry, providing an ensemble of flexible variations for conformational selection or induced-fit binding mechanisms. Overall, this analysis demonstrated, as expected, a large degree of potential motion, with the more disordered portions of the protein flexing more than those with secondary structure that was not apparent from the structural analysis (Figures 4 and 5).

## Discussion

The TCR governs T-cell specificity by discriminating between self and foreign peptides presented by MHC molecules. The finger-like CDR loops of the TCR are thought to meld around specific pMHCs, sampling the peptide cargo, and enabling T-cell triggering by ligands with sufficient affinity/dwell-time. This binding mode is also likely to facilitate TCR cross-reactivity by enabling the TCR to explore multiple conformations during ligand interrogation. However, the mechanism(s) that underpin the ability of T-cells to respond to millions of different pMHCs are still emerging. Several experiments have used atomic resolution structures to compare TCRs in unbound state and in complex with pMHC (23). These studies revealed conformational changes upon binding, supporting the idea that the CDR loops can flex to accommodate different peptide cargos using an induced fit mechanism. More recent data, using NMR, FRET, and MD support this view, but also demonstrate that the TCR-pMHC interface can be far more flexible than is apparent from the static image captured during X-ray crystallography (17–22).

First, we examined a very broad, but unanswered question: Is the conformation of a protein identical in every dataset collected and refined during X-ray crystallography experiments? This question is relevant to all structures solved by X-ray crystallography, but is particularly applicable when investigating ligand engagement by a receptor, as in the case of TCR-pMHC interaction. Several factors could affect the refined structure generated during this approach including; changes in the crystal packing between crystals, alterations in lattice contacts that could artificially stabilize protein regions, interpretation of the data during refinement, and differences in the protein preparation. In order to try to test some of these factors, we solved the structure of the same three TCRs from multiple crystals, grown in a range of conditions, from several different protein preparations, refined by different scientists.

Reassuringly, the overall conformation of each structure was virtually identical in all datasets tested. However, we observed several CDR loop re-organizations between structures for the HA1.7 and F11 TCRs, while all structures of the 003 TCR were identical at the level of the Cα backbone. These observations were not linked to the resolution of the structures, the crystal growing conditions, the availability of stabilizing crystal lattice contacts, or on who performed the refinement. Thus, we conclude that these loop movements represent real differences in the conformation of the CDR loops of the HA1.7 and F11 TCRs due to the intrinsic flexibility of these regions. We, therefore, recommend caution when using comparisons of unbound and pMHC-bound TCRs to describe binding mechanisms as these movements assume that the unbound structure of the TCR is a representative low energy state. Our observations suggest that the snapshot provided by X-ray crystallography may not be representative of CDR loop positions because of the highly dynamic nature of these disordered regions of the TCR.

To further investigate the flexible nature of the CDR loops, we measured large-amplitude protein motions in the three TCRs under investigation using FIRST/FRODA software. As expected, this analysis demonstrated a large degree of potential motion, with the more disordered portions of the protein flexing more than those with secondary structure. This analysis was far more revealing than the structural analysis alone, which only demonstrated structural mobility in some of the loops of just two of the TCRs (F11 and HA1.7). Rather, we observed large potential motions in all of the TCR CDR loops of all three TCRs, with more rigidity detected in the non-CDR loop portions of the TCR. This flexibility, which has been assumed, but not definitively proven for the TCR CDR loops, is consistent with the notion that the mechanism by which the TCR samples pMHC epitopes relies on flexibility at the interaction interface. Finer dissection of the motions of each individual CDR loop (including the Fw loop) demonstrated different maximal amplitudes at the apex of each loop. On average, the 003 TCR CDR loops moved slightly less compared to the F11 and HA1.7 TCR CDR loops, in line with the structural analysis. Furthermore, the somatically rearranged CDR3 loops in all of the TCRs studies were more mobile compared to the germline encoded CDR1, 2, and Fw loops. These findings are consistent with the observation that (1) the CDR3 loops are generally longer than the other loops, and (2) the CDR3 loops generally form the majority of the interactions with the variable peptide cargo, compared to the more MHC-centric CDR1, 2, and Fw loops. Thus, this extra level of flexibility in the CDR3 loops may represent an important mechanism enabling TCR cross-reactivity with multiple different peptides (2, 3, 10, 11).

These data have important implications for the general analysis of crystal structures, and more specifically for TCR antigen recognition. With the recent breakthroughs in MD and other modeling approaches, technologies that will rapidly develop in the near future, it seems rational to start pairing crystal structures with this type of analysis. Although theoretical, modeling approaches can provide another dimension of information to the complex and flexible amino acid network that governs the nature of protein–ligand dynamics. Findings from these analyses may reveal new areas of interest that can be tested experimentally. Our data, demonstrating the theoretical range of motion for unbound TCRs, are consistent with other modeling approaches that have focused on TCR-pMHC complexes (28–33), or TCRs alone (21). These studies have shown that the TCR-pMHC interface is highly flexible with some fixed interactions, but others that come and go as the TCR “rocks” on top of the pMHC (33). This binding mode is also congruous with recent experimental data demonstrating that TCRs are highly degenerate and can recognize many thousands, if not millions, of different peptide sequences (9–13). This enables T-cells to cross-react, thereby allowing a limited pool of TCR sequences within an individual to afford protection against the vast milieu of potential pathogenic peptide sequences that could be encountered (2, 3). Finally, our data reinforce the notion that some TCR CDR loops form a highly flexible and dynamic binding site, and that crystal structures alone may not be adequate to fully represent the complex mechanisms employed during pMHC ligation by the TCR. The fact that CDR loops can “move” between different free TCR structures of the same molecule suggests that caution is advisable when inferring binding mode based on the single snapshots provided by X-ray crystallography of ligated and unligated TCR.

## Author Contributions

PR, SW, and DC interpreted the data and designed experiments. CH, BM, VB, SH, RM, OV, AB, AF, PR, SW, and DC performed experiments. AG, AS, SW, and DC funded the study. AS, SW, and DC wrote the manuscript.

## Conflict of Interest Statement

The authors declare that the research was conducted in the absence of any commercial or financial relationships that could be construed as a potential conflict of interest.
